# Thermosets Based on Covalent Bond Exchange: Mechanisms, Properties, and Reprocessing

**DOI:** 10.3390/polym18111317

**Published:** 2026-05-27

**Authors:** Xiaojuan Shi, Daotong Zhuang

**Affiliations:** 1 Shanghai Institute of Applied Mathematics and Mechanics, Shanghai Key Laboratory of Mechanics in Energy Engineering, School of Mechanics and Engineering Science, Shanghai University, Shanghai 200072, China; 2 Shanghai Institute of Applied Mathematics and Mechanics, Shanghai Key Laboratory of Mechanics in Energy Engineering, Shanghai Frontier Science Center of Mechanoinformatics, School of Mechanics and Engineering Science, Shanghai University, Shanghai 200072, China

**Keywords:** thermosets, covalent adaptable networks, bond exchange mechanisms, reprocessability

## Abstract

Thermosets are widely used in engineering applications due to their high mechanical strength, thermal stability, and chemical resistance; however, their permanently crosslinked networks also limit repair, reshaping, and recycling. Dynamic covalent chemistry offers a route to addressing these limitations through the incorporation of reversible bond exchange into thermoset networks. A range of dynamic thermosets has been developed based on transesterification, Diels–Alder reactions, imine exchange, disulfide metathesis, boronic ester exchange, and siloxane equilibration, enabling self-healing, reprocessing, welding, and closed-loop recycling. This review examines representative dynamic thermosets in terms of exchange mechanisms, network topology evolution, and macroscopic response. By correlating molecular exchange processes with network-level mechanics and macroscopic performance, this review identifies design principles for dynamic thermosets with improved sustainability and processing compatibility.

## 1. Introduction

Thermosets are valued for their high mechanical rigidity, thermal stability, chemical resistance, and dimensional stability, all of which originate from their permanently crosslinked network structures [1,2,3,4]. These properties underpin their use across a wide range of applications, including aerospace composites, electronic encapsulation, structural adhesives, coatings, and fiber-reinforced materials [5,6,7]. However, the permanent covalent crosslinks in conventional thermosets impose a key limitation—once cured, these materials cannot be reshaped, repaired, or efficiently recycled [8]. With the increasing global production and use of thermosetting polymers, their limited recyclability is becoming an growing environmental concern [9,10,11].

Dynamic covalent chemistry provides a route to addressing these limitations [12,13]. Dynamic covalent bonds undergo reversible formation and cleavage under specific conditions, enabling molecular rearrangement while retaining covalent bond strength [14]. In contrast to supramolecular systems based on non-covalent interactions, dynamic covalent networks retain structural stability while permitting reversible rearrangement [15]. This reversibility enables networks to reorganize toward thermodynamically favored structures [16,17]. Incorporation of dynamic covalent bonds into thermosets has led to covalent adaptable networks (CANs), which can exhibit self-healing, reprocessability, closed-loop recyclability, and stimuli-responsive behavior [18,19,20,21]. Representative dynamic chemistries include transesterification, Diels–Alder reactions, imine exchange, disulfide metathesis, boronic ester exchange, and siloxane equilibration [22,23,24,25,26,27]. Despite these advances, dynamic covalent bonds do not simply confer recyclability as a universal property. Different exchange chemistries instead produce distinct modes of network topology evolution, which govern stress relaxation, creep resistance, dimensional stability, and recycling pathways [28,29]. Exchange kinetics, reaction extent, network mobility, and local chemical environment together determine whether bond exchange leads to macroscopic flow, partial rearrangement, or complete depolymerization, while also influencing processing temperature, catalyst requirement, solvent involvement, and the overall sustainability of recycling and reprocessing processes [30,31,32]. Previous reviews of dynamic thermosets and covalent adaptable networks have mainly emphasized specific dynamic chemistries, self-healing, or recycling approaches. In this review, the discussion is organized around the relationship between the exchange pathway, network topology evolution, stress relaxation, and reprocessability.

This review does not aim to comprehensively summarize all reversible chemistries or adaptive polymer systems. Supramolecular networks based solely on non-covalent interactions, linear dynamic polymers without permanent cross-linked structures, and dynamic chemistries that have seen limited application in thermosetting networks are not discussed in detail. Representative dynamic thermosetting networks based on transesterification, Diels–Alder reactions, imine exchange, disulfide metathesis, boronic ester exchange, and siloxane equilibration are discussed. These systems cover associative, dissociative, and equilibrium-controlled exchange pathways while also representing different sensitivities to catalysts, neighboring-group effects, environmental conditions, and chain mobility. Through comparisons of representative exchange mechanisms and their corresponding network behavior, this review highlights how molecular exchange processes govern macroscopic performance and reprocessability in dynamic thermosets.

## 2. Covalent Bond Exchange Mechanisms

**Figure 1:**
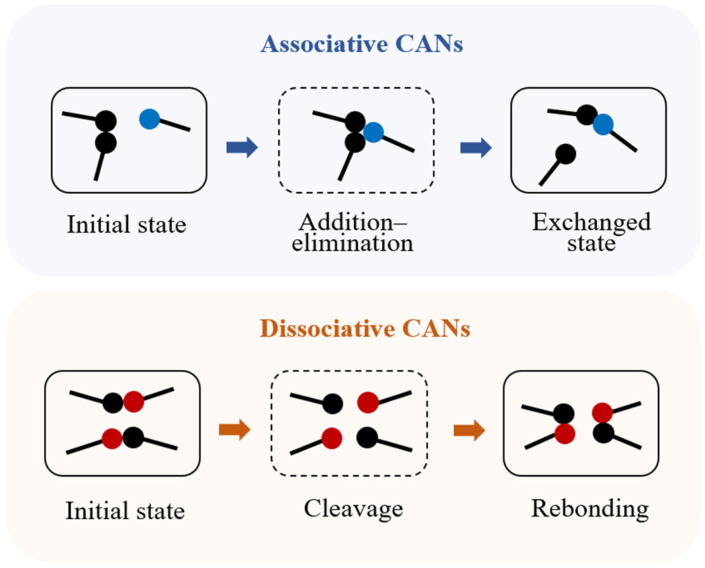
Schematic illustration of associative and dissociative bond exchange mechanisms in CANs.

Dynamic bond exchange in thermosets can be broadly classified as associative or dissociative. Figure 1 illustrates associative exchange proceeding via an addition–elimination pathway, where bond formation precedes bond cleavage, whereas dissociative exchange involves bond cleavage prior to reformation into new crosslinks [12,33]. The key distinction between these pathways lies in how network connectivity evolves during rearrangement [34]. Associative exchange can enable efficient stress relaxation while largely preserving network connectivity, which helps limit permanent deformation and maintain dimensional stability during service [35]. In contrast, dissociative exchange may transiently reduce cross-link density during bond cleavage, increasing chain mobility and promoting creep or plastic flow under prolonged thermal or mechanical loading [36]. Recent studies indicate that the macroscopic consequences of these pathways are not always distinct [37]. Even in dissociative systems, only a limited fraction of bonds is cleaved at a given time, so the overall network remains largely intact during reprocessing. As a result, dissociative networks can exhibit vitrimer-like rheological behavior over certain temperature and timescales. This suggests that network topology evolution depends not only on the exchange mechanism but also on the extent and kinetics of bond dissociation [38].

In addition to their intrinsic exchange mechanism, the dynamic behavior of thermosets is governed by structural and environmental factors [39]. Catalyst identity and loading influence exchange kinetics, whereas internal catalysis and neighboring-group participation can enable catalyst-free or self-activated pathways [40,41]. The local chemical environment, including solvents and small-molecule additives, can modulate reaction pathways, particularly in systems involving dissociative exchange or hydrolysis–reformation processes [42,43]. Network characteristics are also critical, as crosslink density and network architecture determine the number of elastically active chains and influence bond exchange kinetics, whereas the glass transition temperature (*T*_g_) and chain mobility govern whether exchange reactions translate into macroscopic flow or relaxation [44]. Swelling or reactive solvents may also enhance chain mobility or directly participate in bond exchange, altering both kinetics and mechanism [22]. These factors influence how network connectivity and chain mobility change during bond exchange and therefore affect stress relaxation and macroscopic viscoelastic behavior [45]. From a viscoelastic perspective, stress relaxation in dynamic thermosets can be described as the collective relaxation of elastically active network chains with different characteristic relaxation times [46,47]. In heterogeneous dynamic networks, variations in exchange environment, catalyst distribution, or network structure can contribute differently to the overall relaxation spectrum, often producing non-single-exponential relaxation behavior [48]. Generalized Maxwell-type models are therefore often used to relate molecular exchange processes to macroscopic viscoelastic response and to connect rheological relaxation with exchange kinetics, network rearrangement, and chain mobility [48,49].

These mechanistic and viscoelastic differences also influence processing, recycling, and the practical use of dynamic thermosets. Compared with conventional thermoset disposal, pyrolysis, or high-temperature chemical recycling, some dynamic covalent networks can be reprocessed under milder conditions with lower energy input during material recovery [50]. However, many systems still require elevated temperatures, catalysts, or solvent-assisted depolymerization, which can increase processing complexity and overall environmental or economic cost [31]. In addition, catalyst selection and loading can substantially influence processing efficiency and recycling conditions, whereas solvent-assisted recycling may further complicate solvent recovery and purification during reprocessing [31,51]. Repeated processing can also gradually change network structure and material performance as catalyst deactivation or leaching, oxidative degradation, hydrolysis, and side reactions may progressively reduce crosslink density and weaken mechanical properties after multiple recycling cycles [52]. As a result, improving reprocessability or recycling efficiency may also compromise long-term mechanical integrity, dimensional stability, or thermal robustness under repeated processing. These effects are especially important in structural and composite applications, where dimensional stability, creep resistance, and long-term durability must be retained during service [15,53]. Practical use also depends on compatibility with existing manufacturing methods such as compression molding, extrusion, injection molding, resin transfer molding, and composite fabrication [54,55]. Differences in exchange kinetics, chain mobility, and network rearrangement can therefore lead to substantial variation in processing behavior and long-term stability among dynamic thermosets. To facilitate comparison among representative dynamic thermosetting networks, Table 1 summarizes key mechanistic and environmental factors governing topology evolution and macroscopic behavior in representative dynamic thermosetting networks.

Representative dynamic thermosets also differ substantially in terms of their operating temperatures and relaxation timescales. Differences in activation energy and stress relaxation behavior influence both the rate of network rearrangement and the processing conditions required for reprocessing or recycling. Table 2 summarizes representative activation-energy ranges together with typical processing temperature windows for major classes of dynamic thermosets.

## 3. Thermosets Based on Representative Dynamic Covalent Reactions

Dynamic covalent reactions have been incorporated into thermosetting polymer networks to enable reversible bond exchange and network rearrangement. These reactions proceed through mechanisms such as reversible addition–elimination, metathesis, or condensation, allowing for network rearrangement under external stimuli, including heat, light, or chemical triggers [35]. Building on the mechanistic framework outlined in Section 2, representative dynamic chemistries in covalent adaptable thermosets include transesterification, Diels–Alder reactions, imine exchange, disulfide metathesis, boronic ester exchange, and siloxane equilibration. Despite their distinct molecular mechanisms, these dynamic bonds enable network topology rearrangement in crosslinked networks and give rise to macroscopic properties such as repairability, recyclability, and malleability.

### 3.1. Transesterification-Based Thermosets

**Figure 2:**
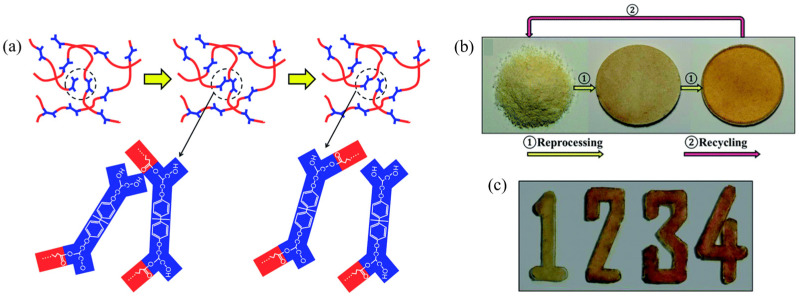
(**a**) Representative associative transesterification bond exchange mechanism showing structural evolution of active functional groups; (**b**) closed-loop reprocessing and recycling of transesterification-based covalent networks; and (**c**) preserved shape fidelity over multiple cycles [58]. Copyright 2014 The Royal Society of Chemistry.

Transesterification is a widely used bond exchange mechanism in covalent adaptable networks, enabled by the synthetic accessibility and structural versatility of ester linkages across diverse polymer backbones [119]. In 2011, Leibler and co-workers established transesterification in epoxy-based vitrimers as a thermally activated associative exchange process, in which network topology rearranges while the overall crosslink density is preserved [56]. Subsequent studies showed that catalyst identity and loading govern exchange kinetics and the topology-freezing transition, and that metal-catalyzed transesterification enables welding and healing via interfacial bond exchange [26,57]. Qi and co-workers further showed that network stoichiometry and glass transition temperature (*T*_g_) influence stress relaxation, establishing a quantitative relationship between network rigidity and exchange kinetics [59]. Figure 2 illustrates both the associative transesterification bond exchange mechanism and the resulting closed-loop reprocessing behavior of transesterification-based covalent networks [58]. Repeated reprocessing can largely preserve bulk integrity and shape fidelity, although gradual changes in modulus, glass transition behavior, and ultimate stretch may still occur after multiple cycles because permanently crosslinked strands that have fractured during pulverization cannot be completely recovered during subsequent bond exchange and healing processes.

**Figure 3:**
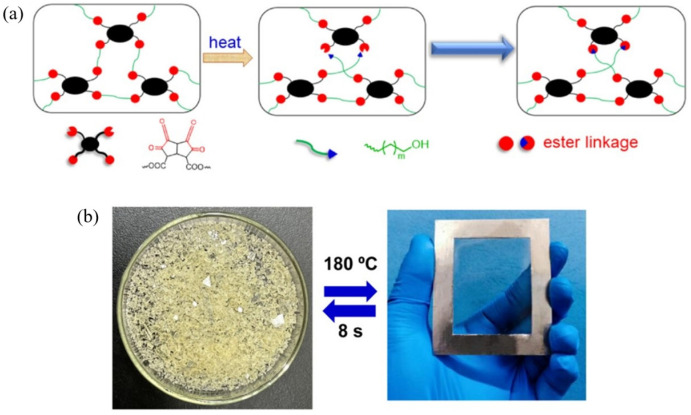
Ultrafast reprocessing enabled by dissociative transesterification in β-ketoester networks. (**a**) Dissociative transesterification-induced topology rearrangement; (**b**) ultrafast reprocessing within seconds [64]. Copyright 2024 American Chemical Society.

Transesterification is not limited to a single associative mechanism. In 2019, Du Prez and co-workers reported phthalate monoester networks in which transesterification is internally catalyzed by a neighboring carboxylic acid and proceeds through a dissociative pathway involving a cyclic anhydride intermediate [40]. Despite this dissociative mechanism, the materials exhibit Arrhenius-type stress relaxation and vitrimer-like rheology under typical conditions while undergoing gel–sol transitions in hot swelling solvents. Rheological behavior alone cannot uniquely define the underlying exchange pathway. A similar mechanistic ambiguity is observed in phosphate triester systems, where catalyst-free transesterification, which is likely to be associative or concerted, produces vitrimer-like flow but permits decrosslinking under specific conditions [63]. Dissociative transesterification has also been developed as a distinct design paradigm. In 2024, Pang and co-workers reported β-ketoester-based polyester networks that undergo catalyst-free transesterification via a unimolecular dissociative pathway involving acylketene intermediates [64]. This mechanism enables ultrafast stress relaxation (τ ≈ seconds) and reprocessing within seconds, which is attributed to transient reductions in crosslink density during exchange (Figure 3). This strategy was extended to diethyl 1,3-acetonedicarboxylate-based systems, enabling reprocessing under milder conditions (minutes at 150 °C) and efficient closed-loop recycling via selective depolymerization [65].

These transesterification systems show that vitrimer-like stress relaxation and reprocessability do not necessarily arise from the same exchange mechanism. Associative transesterification typically preserves network connectivity during exchange, making it more suitable for applications requiring dimensional stability, creep resistance, and retention of crosslink density during reprocessing [59,135,136,137]. By contrast, internally catalyzed dissociative systems enable catalyst-free exchange through neighboring-group participation, but their behavior is often more sensitive to the solvent environment, swelling, and temporary decrosslinking during rearrangement [40,138]. Catalyst-free dissociative systems further show that ultrafast relaxation and closed-loop depolymerization are possible without external catalysts, although temporary loss of network connectivity can reduce high-temperature stability and dimensional integrity [63,64,139,140]. These differences show that the performance of transesterification-based thermosets depends not only on exchange kinetics, but also on how network connectivity, the catalyst environment, chain mobility, and processing conditions evolve during bond exchange.

### 3.2. Diels–Alder Thermoreversible Thermosets

**Figure 4:**
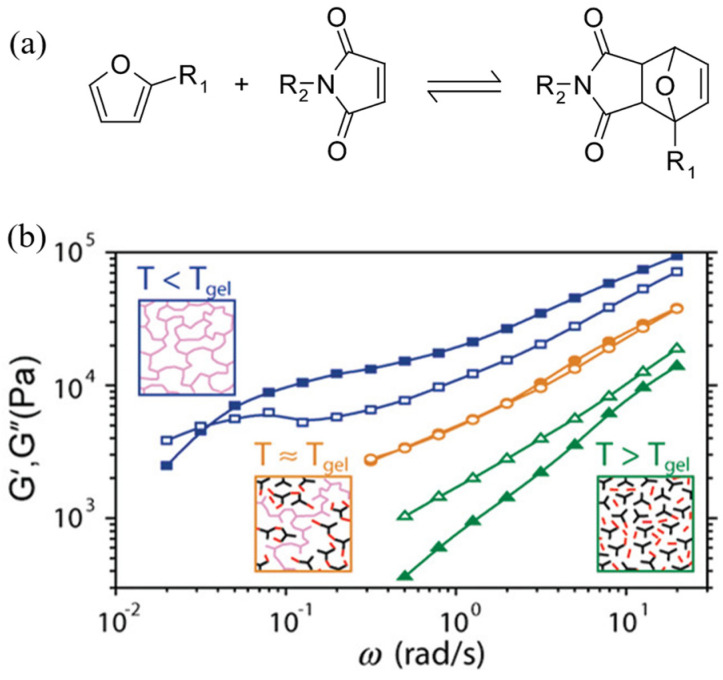
(**a**) Thermoreversible Diels–Alder/retro-Diels–Alder cycloaddition between furan and maleimide motifs; (**b**) frequency-dependent rheological response showing modulus crossover and sol–gel transition in DA-based networks [143]. Copyright 2008 American Chemical Society.

Diels–Alder (DA) chemistry is a representative dynamic covalent platform characterized by a thermally reversible equilibrium between cycloaddition and retro-Diels–Alder (rDA) cleavage [121]. Owing to the high chemoselectivity, catalyst-free bond formation, and reversible equilibrium, DA reactions have been widely used to construct thermoreversible thermosets with programmable network dynamics [141]. Wudl and co-workers established furan/maleimide networks as repairable crosslinked polymers, in which DA adducts dissociate upon heating and reform upon cooling, enabling repeated crack healing without external agents [142]. Lehn and co-workers demonstrated that appropriately designed DA pairs can reach thermodynamic equilibrium even at ambient conditions, indicating that DA bonds function as equilibrium-controlled dynamic linkages rather than merely thermally cleavable units [68]. Bowman and co-workers provided a quantitative framework showing that DA equilibrium conversion is strongly temperature-dependent and governs gel–sol transitions, with a defined gel-point temperature and reversible network rearrangement reflected in rheological relaxation (Figure 4) [143]. These results reveal a direct coupling between bond equilibrium and macroscopic viscoelasticity, establishing DA chemistry as a model system for temperature-programmed network reversibility. DA linkages have also been incorporated into functional materials to extend their response modes. In elastomeric matrices, retro-DA cleavage induces solid-to-liquid transitions and serves as an intrinsic thermal energy dissipation pathway, while enabling reprocessability [144]. Repeated thermal cycling, however, can lead to competing irreversible side reactions that gradually reduce network reversibility and healing efficiency. Alternative motifs, such as hetero-Diels–Alder systems, can overcome kinetic limitations by enabling faster exchange rates and efficient self-healing under milder conditions, thereby expanding the accessible temperature window [145].

**Figure 5:**
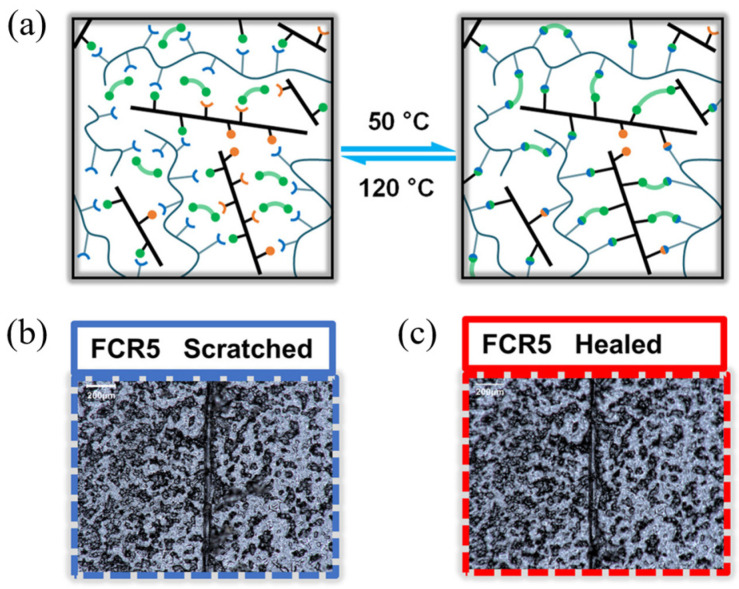
(**a**) Schematic illustration of the DA crosslinked network showing reversible bonding at 50 °C and debonding at 120 °C. Optical images of scratched surfaces (**b**) before and (**c**) after thermal healing in nanocomposites with 5% CNT loading [146]. © 2026 SPE-Inspiring Plastics.

DA chemistry has been integrated into structurally complex and multifunctional systems. Incorporation of photothermal fillers, magnetic particles, or polyurethane matrices enables dynamic regulation of crosslink density under external stimuli, resulting in materials that combine self-healing, recyclability, and stimulus-responsive mechanical behavior [75,76]. A fundamental structure–property trade-off is observed: increasing DA crosslink density improves mechanical strength (e.g., in high-performance adhesives) but often reduces repeated healing efficiency due to restricted chain mobility and accumulation side reactions [77]. In nanocomposite systems, reversible bonding at the polymer–filler interface is important because interfacial regions often experience stress concentration and restricted chain mobility during deformation and damage. Diels–Alder chemistry is well suited to these systems because DA bonds can reversibly dissociate and reform at the polymer–CNT interface, allowing interfacial adhesion to recover during healing and reprocessing. In CNT-filled thermosets, such dynamic interfacial interactions can promote stress redistribution and crack repair while preserving mechanical reinforcement [146]. Figure 5 illustrates this behavior, where thermally reversible DA crosslinks enable interfacial bonding and debonding, leading to effective scratch healing in CNT-filled nanocomposites.

Although Diels–Alder thermosets exhibit excellent thermoreversible processability, their dynamic behavior is governed by equilibrium-controlled bond dissociation rather than associative bond exchange. At elevated temperatures, retro-Diels–Alder reactions progressively decrease crosslink density, which can promote partial gel–sol transition, creep, and loss of dimensional stability [143,147]. The equilibrium responsible for healing and reprocessing can therefore also reduce thermomechanical stability during prolonged heating or repeated processing cycles. Repeated heating and cooling can also result in incomplete reversibility, side reactions, and gradual loss of mechanical performance, depending on the DA pair, processing temperature, and network structure [121]. Diels–Alder thermosets are therefore well suited to reversible reshaping and mild reprocessing, but the equilibrium-driven decrease in network connectivity can limit long-term stability at elevated temperatures.

### 3.3. Imine Exchange Thermosets

**Figure 6:**
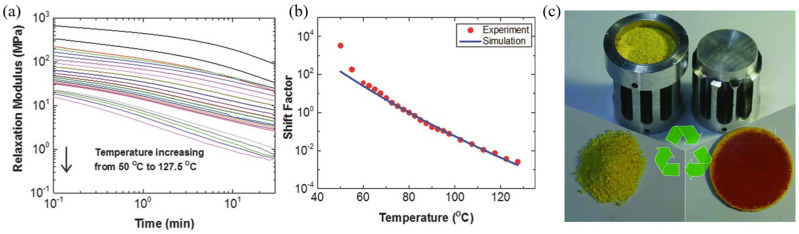
(**a**) Stress relaxation behavior of polyimine networks at different temperatures; (**b**) time–temperature superposition behavior of polyimine thermosets; and (**c**) repeated reprocessing of imine-linked polymers from powder to bulk solids [22]. Copyright 2014 Wiley-VCH GmbH, Weinheim.

Imine exchange is a condensation-based dynamic covalent platform characterized by synthetic simplicity, catalyst-free exchange, and chemical addressability [78]. Imine formation and cleavage are reversible, allowing for these networks to be constructed via aldehyde–amine condensation [54]. Dynamic bond exchange proceeds primarily through transamination, often without external catalysts and under mild thermal or chemical conditions [79]. In 2014, Zhang and co-workers established polyimine thermosets as catalyst-free dynamic networks that behave as conventional glassy materials under ambient conditions but exhibit Arrhenius-type stress relaxation and reprocessability upon heating [22]. Figure 6 shows the thermally activated stress relaxation behavior, time–temperature superposition response, and repeated powder-to-bulk reprocessing of polyimine networks. These results illustrate that imine exchange thermosets can combine vitrimer-like reconfigurability with relatively stable network integrity during repeated processing.

**Figure 7:**
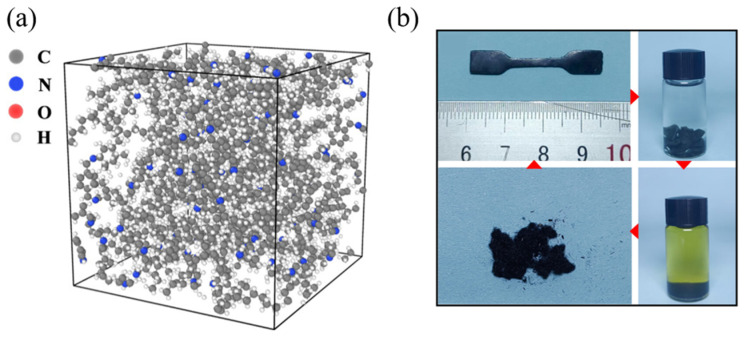
(**a**) All-atom MD model of the biobased polyimine elastomer; (**b**) closed-loop recyclable process of the polyimine–carbon-fiber composite [86]. Copyright 2026 American Chemical Society.

Imine exchange has been extended to hierarchically structured systems, including interfaces and composites. In elastomer/graphene vitrimer systems, imine exchange enables dynamic bonding within the polymer matrix and at the polymer–filler interface, resulting in temperature-activated flow, infrared-triggered reshaping, and stress relaxation governed by chain constraints and the availability of free amine groups [84]. Imine chemistry can be integrated into more rigid backbones, as demonstrated in polyimide-derived thermosets that retain high tensile strength and elevated *T*_g_ while exhibiting rehealability and recyclability [85]. Biobased polyimine elastomers and fiber-reinforced composites combine room-temperature self-healing, rapid stress relaxation, and closed-loop chemical recycling under mild acid/base conditions, as supported by both experiments and molecular-level simulations [86]. Figure 7 illustrates this behavior through combined molecular modeling and macroscopic recycling performance.

Transamination provides efficient, often catalyst-free bond exchange, whereas imine linkages are inherently susceptible to hydrolysis and chemical conditions, particularly in the presence of water or under acidic conditions [39]. Thermal reprocessability and chemical recyclability are therefore often simultaneously enabled in imine-based networks, but at the cost of increased environmental sensitivity. This duality distinguishes imine chemistry from more kinetically stable dynamic bonds and necessitates a balance between exchange kinetics and network stability through molecular design and environmental control.

### 3.4. Disulfide Exchange Thermosets

**Figure 8:**
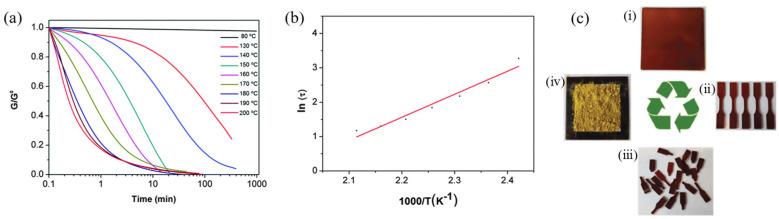
(**a**) Stress relaxation behavior at different temperatures; (**b**) Arrhenius analysis of stress relaxation kinetics; and (**c**) repeated reprocessing of disulfide-crosslinked epoxy vitrimers: a pristine film (**i**) was fabricated into dumbbell-shaped specimens (**ii**), followed by tensile testing (**iii**), powder grinding (**iv**), and hot pressing to regenerate recycled films [149]. Copyright 2016 The Royal Society of Chemistry.

Disulfide exchange is a versatile dynamic covalent platform characterized by fast exchange kinetics, multiple activation modes, and efficient interfacial healing capability [96,97]. Disulfide-based networks were initially developed in self-healing elastomeric and rubber systems, where dynamic S–S exchange enables efficient restoration of damaged interfaces. In 2011, Klumperman and co-workers showed that incorporating disulfide linkages into covalently crosslinked rubber networks enables autonomous healing of macroscopic cuts at moderate temperature, with near-complete recovery of elongation at break after repeated cycles [23]. Disulfide metathesis can be catalytically accelerated; for example, Zhang and co-workers reported that CuCl_2_-enabled disulfide exchange in vulcanized rubber through a complex-mediated, non-radical pathway, facilitates crack healing and material recycling in industrially relevant systems [148]. In 2016, Odriozola and co-workers incorporated aromatic disulfide exchange into high-*T*_g_ epoxy thermosets and observed vitrimer-like stress relaxation above *T_g_* while maintaining mechanical robustness during repeated processing cycles [149]. Figure 8 shows the thermally activated stress relaxation behavior and repeated reprocessing of aromatic disulfide-crosslinked epoxy vitrimers.

**Figure 9:**
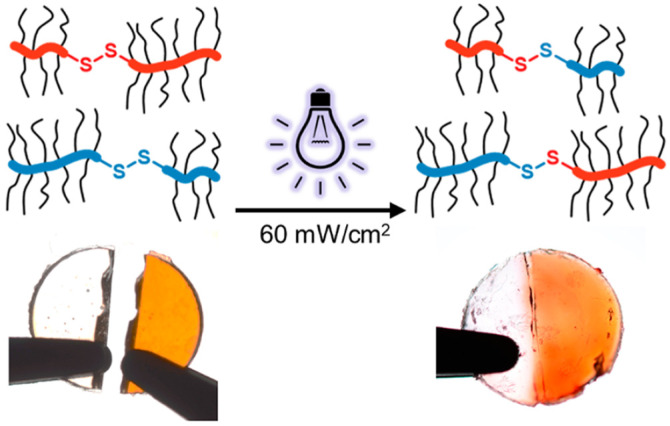
Light-induced self-healing of disulfide-based dynamic materials via reversible S–S bond exchange [98]. Copyright 2021 American Chemical Society.

Disulfide-based systems have advanced toward industrially relevant processing and high-performance recyclable materials. In soft materials, light-mediated disulfide exchange enables catalyst-free network formation and self-healing under ambient conditions [98]. Figure 9 shows self-healing enabled by disulfide exchange under photochemical conditions. Torkelson and co-workers developed disulfide-based crosslinkers with enhanced structural definition, which enable rapid stress relaxation, complete recovery of crosslink density, and melt extrusion processing at elevated temperatures [150]. This strategy was extended to polyethylene-based covalent adaptable networks synthesized via high-pressure free-radical copolymerization, which yield crosslinked materials with high crystallinity, dimensional stability, and full recyclability [151]. Multi-pathway dynamic networks have been developed to address the trade-off between mechanical robustness and recyclability. Sulfur-rich episulfide thermosets incorporating both sulfhydryl–disulfide exchange and disulfide–disulfide metathesis exhibit reduced activation energy for topology rearrangement (~33–34 kJ mol^−1^) while maintaining high *T*_g_, mechanical strength, rapid self-healing, and near-complete chemical recyclability under mild conditions [152].

These disulfide systems show that exchange kinetics and macroscopic behavior in disulfide thermosets depend strongly on both disulfide structure and activation pathway. Aromatic disulfides generally undergo faster exchange because aryl substitution stabilizes thiyl radical intermediates and lowers the activation barrier for bond exchange [153,154,155]. Aromatic disulfide thermosets therefore often exhibit efficient stress relaxation, room-temperature self-healing, and facile reprocessing, but can also become more susceptible to creep and unintended network rearrangement during prolonged thermal exposure [156]. By contrast, aliphatic disulfides typically require stronger thermal, photochemical, redox, or catalytic activation to achieve efficient exchange, which can improve dimensional stability and suppress spontaneous network rearrangement during service [97,157]. These differences show that the performance of disulfide-based thermosets depends not only on exchange rate, but also on how activation pathway, radical stability, and network mobility influence creep and long-term structural relaxation.

### 3.5. Boronic Ester-Based Thermosets

**Figure 10:**
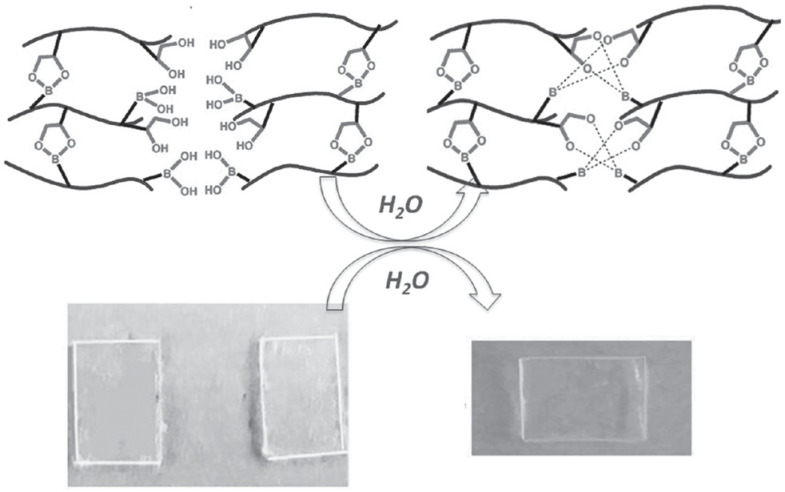
Proposed mechanism and room-temperature self-healing behavior of boronic ester-based elastomer networks in air [158]. Copyright 2016 Wiley-VCH GmbH, Weinheim.

Boronic ester-based thermosets are a class of dynamic covalent networks in which bond exchange kinetics are tunable and strongly influenced by the local chemical environment [106]. Boronic ester exchange generally proceeds through associative pathways that preserve network connectivity during topology rearrangement [42]. In 2015, Guan and co-workers showed that the rate of boronic ester exchange can be modulated over orders of magnitude through neighboring-group effects, and that these kinetic differences translate into bulk malleability and self-healing efficiency in polymer networks [41]. Dynamic behavior in boronic ester systems can be programmed through local chemical structures. These networks can exhibit room-temperature self-healing in elastomeric materials, indicating that efficient bond exchange can occur under mild conditions without elevated temperatures or external catalysts [158]. Figure 10 demonstrates the proposed boronic ester exchange mechanism together with the corresponding room-temperature self-healing behavior observed in boronic ester-based elastomer networks.

**Figure 11:**
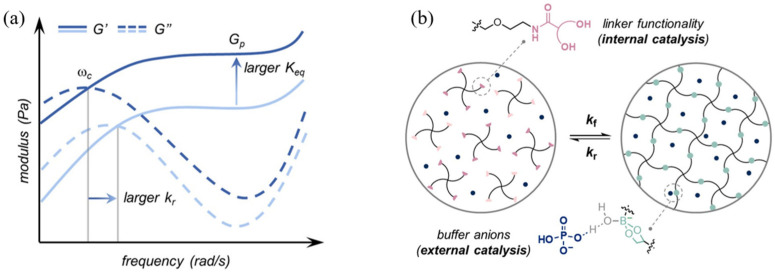
(**a**) Schematic illustration of dissociative exchange-controlled viscoelasticity and frequency-dependent network response; (**b**) proposed internal amide-assisted and buffer anion-mediated catalytic pathways in boronic ester exchange networks [107]. Copyright 2022 American Chemical Society.

A defining feature of boronic ester networks is the role of internal and external catalysis mediated by the chemical microenvironment. Kalow and co-workers showed that exchange kinetics are not only determined by the diol–boronic acid pair itself, but also influenced by proximal functional groups (e.g., amides) and buffering anions, which act as internal and external catalysts [107]. Figure 11 illustrates the effects of internal and external catalysis on exchange kinetics and viscoelastic behavior in boronic ester dynamic networks, highlighting how the local chemical environment regulates both molecular exchange and macroscopic network response. This coupling between bond exchange and the local environment indicates that macroscopic properties arise from the interplay of bond thermodynamics, exchange kinetics, and chemical surroundings, rather than bond reversibility alone. In vitrimeric and photocurable systems, tuning dioxaborolane structure and concentration controls polymerization behavior, thermomechanical properties, and the balance between malleability and mechanical strength [108]. In structural materials, incorporation of secondary interactions such as B–N coordination enhances mechanical robustness while preserving dynamic functionality [159]. Boronic ester exchange has been implemented in epoxy-based thermosets, where reversible topology rearrangement enables both physical reprocessing and chemical degradation while maintaining key functional properties, including dielectric strength and thermal stability [160].

Compared with transesterification- or disulfide-based systems, boronic ester thermosets are more sensitive to the local chemical environment because hydrolysis, esterification equilibrium, and exchange kinetics can all be affected by pH, humidity, buffering species, and neighboring-group interactions [99]. Environmental conditions influence both the exchange rate and network stability. Studies on neighboring-group effects and internal or external catalysis further show that small changes in the local chemical environment can substantially alter viscoelastic responses, stress relaxation, and healing behavior [161]. In aqueous environments, local ionic interactions and alkaline regulation can stabilize boronic ester bonds, thereby extending the accessible pH window and improving underwater healing and recycling performance [162]. Such environmentally sensitive exchange behavior facilitates self-healing and network rearrangement under mild conditions, but it can also reduce dimensional stability under aqueous or hydrolytically active environments.

### 3.6. Siloxane Exchange Thermosets

**Figure 12:**
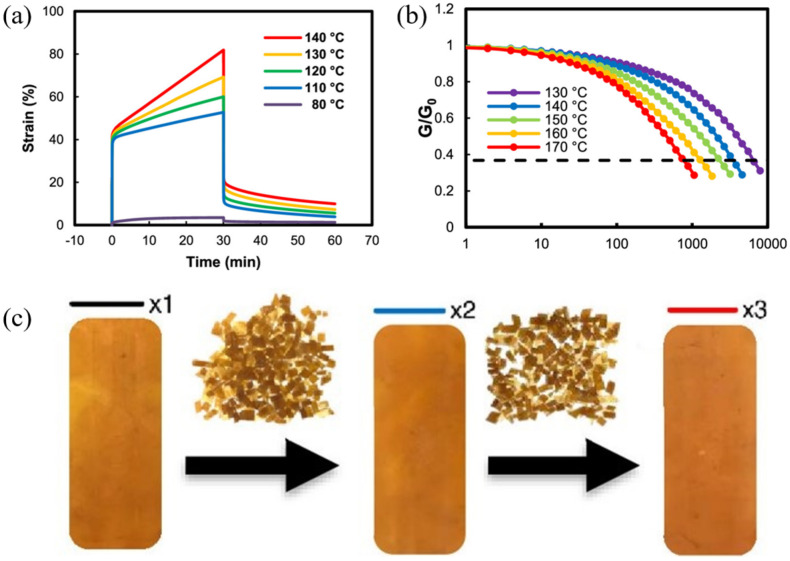
(**a**) Creep behavior of hydroxyl-free silyl ether vitrimer networks at different temperatures; (**b**) thermally activated stress relaxation behavior; and (**c**) repeated grinding/compression-molding reprocessing over multiple cycles [115]. Copyright 2019 American Chemical Society.

Siloxane-based thermosets are dynamic covalent networks characterized by high bond energy, thermal stability, and associative exchange capability, enabling network rearrangement without compromising structural integrity [114]. These features enable applications requiring both thermal robustness and reprocessability. In 2012, Zheng and McCarthy showed that crosslinked PDMS networks bearing silanolate chain ends behave as “living” silicone networks, undergoing thermally activated siloxane equilibration that enables complete healing and repeated reshaping [25]. Si–O bond exchange provides a viable mechanism for dynamic network rearrangement in highly stable silicone systems. This chemistry has been translated into vitrimer design through silyl ether linkages, where exchange kinetics can be modulated through neighboring-group effects. Guan and co-workers showed that intramolecular amine assistance accelerates silyl ether exchange by orders of magnitude, governing stress relaxation and the topology-freezing temperature while preserving thermal robustness [113]. Hydroxyl-dependent exchange pathways present limitations that have been addressed in subsequent designs. Hydroxyl-free silyl ether metathesis enables direct exchange under anhydrous conditions in the presence of Brønsted or Lewis acids, eliminating the need for free hydroxyl groups that can compromise stability at elevated temperatures [115]. Figure 12 shows the creep behavior, stress relaxation, and repeated grinding/compression-molding reprocessing of hydroxyl-free silyl ether vitrimer systems, illustrating their applicability in high-temperature reconfigurable thermosets.

**Figure 13:**
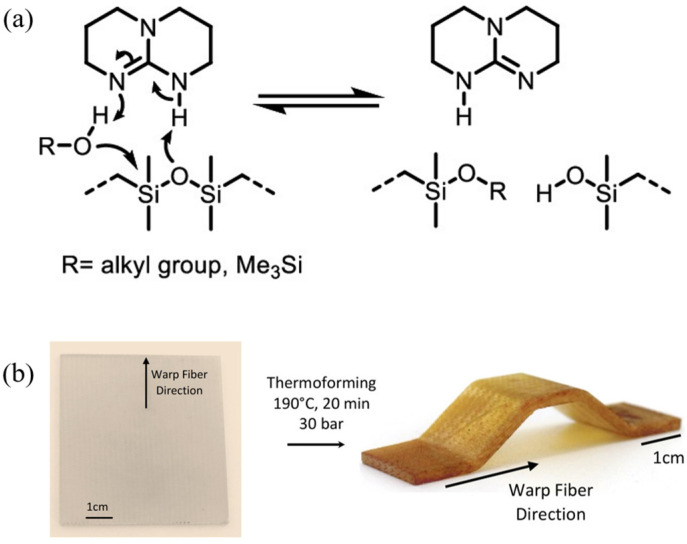
(**a**) TBD-catalyzed siloxane exchange mechanism illustrating base-mediated Si–O bond rearrangement; (**b**) thermoforming behavior of a fiber-reinforced siloxane-containing vitrimer composite [116]. Copyright 2022 American Chemical Society.

Strategies have been developed to accelerate exchange kinetics and expand processing capabilities. TBD-catalyzed siloxane exchange enables ultrafast stress relaxation in epoxy vitrimers while maintaining associative network character. The resulting low-viscosity precursors allow for fabrication of fiber-reinforced composites that can be thermoformed into new shapes (Figure 13) [116]. Balancing dynamic adaptability with dimensional stability leads to silyl ether-crosslinked PDMS elastomers that exhibit near-complete property retention after multiple reprocessing cycles, along with thermal stability and suppressed creep [163]. Internally catalyzed siloxane exchange via amide functionalities enables catalyst-free associative CANs with tunable crosslink density, accelerated stress relaxation, and full recovery of network integrity after reprocessing [117]. The second-order nature of associative siloxane exchange leads to an unusual effect whereby increasing crosslink density enhances exchange kinetics, enabling melt extrusion of highly crosslinked networks. This behavior distinguishes siloxane systems from many other dynamic covalent networks, where increased crosslink density typically suppresses mobility.

Siloxane exchange thermosets are particularly suited to applications requiring thermal stability, dimensional stability, and retention of network integrity at elevated temperatures [114]. Their dynamic behavior is strongly governed by catalyst structure, the exchange activation pathway, and the local chemical environment because siloxane equilibration often relies on nucleophilic or bifunctional catalytic activation involving silanol, hydroxyl, or latent catalytic species [134,164]. Recent studies further demonstrate that fast siloxane exchange can enable efficient reshaping and reprocessing while simultaneously increasing the risk of creep and unintended plastic flow under service conditions [165]. Consequently, the practical performance of siloxane-based dynamic networks depends not only on exchange kinetics, but also on the balance between catalyst activity, thermal stability, and the suppression of creep at elevated temperatures. Thermally reversible or dormant catalyst systems therefore provide an important strategy for expanding the processing window while preserving high-temperature dimensional stability and network integrity.

## 4. Summary

This review summarizes advances in thermosetting polymers enabled by dynamic covalent bond exchange, including transesterification, Diels–Alder reactions, imine exchange, disulfide metathesis, boronic ester exchange, and siloxane equilibration. Despite their mechanistic diversity, these systems enable reversible rearrangement of covalent crosslinks under external stimuli, imparting repairability, recyclability, and malleability to otherwise permanent thermosets. At the molecular level, associative and dissociative exchange pathways govern network topology evolution and macroscopic viscoelastic behavior. Key parameters such as exchange kinetics, crosslink density, the catalyst environment, and network architecture affect stress relaxation, creep resistance, dimensional stability, and processing behavior. Dynamic thermosets have evolved from simple self-healing systems into multifunctional platforms capable of closed-loop recycling, interfacial welding, and recyclable composite and advanced manufacturing. Increasing emphasis has been placed on coupling molecular exchange mechanisms with hierarchical structures and external stimuli to achieve precise property control. Future work should balance mechanical robustness with rapid exchange kinetics, enable low-energy or ambient condition reprocessing, and advance predictive design through deeper mechanistic understanding across molecular, network, and macroscopic scales. These directions will support broader implementation of dynamic covalent thermosets in sustainable and high-performance applications.

## Figures and Tables

**Table 1:** Comparative features governing topology evolution and macroscopic behavior in representative dynamic thermosetting networks.

Dynamic Chemistry	Representative Exchange Pathway	Catalyst Dependence	Neighboring-Group/Internal Catalysis	Solvent/Environmental Sensitivity	Role of Chain Mobility	Typical Topology Evolution Behavior	Ref.
Transesterification	Associative or dissociative depending on system	Often catalyst-mediated; can be catalyst-free	Strong in internally catalyzed monoester systems	Swelling solvents may induce decrosslinking	Governs vitrimer-like relaxation and welding efficiency	Preserved connectivity or transient decrosslinking	[26,56,57,58,59,60,61,62,63,64,65]
Diels–Alder	Equilibrium-controlled reversible cycloaddition	Generally catalyst-free	Limited	Strongly temperature dependent	Controls reversible gel–sol transition	Reversible crosslink dissociation/reformation	[66,67,68,69,70,71,72,73,74,75,76,77]
Imine exchange	Transamination/Hydrolysis–reformation	Usually catalyst-free	Moderate	Sensitive to water and acidic/basic conditions	Influences stress relaxation and healing efficiency	Dynamic rearrangement with hydrolysis susceptibility	[78,79,80,81,82,83,84,85,86,87,88]
Disulfide exchange	Associative radical or metathesis-type exchange	Thermal, photochemical, redox, or catalytic activation	Depends on aromatic/ aliphatic structure	Moderate oxidative/ environmental sensitivity	Strongly affects creep and relaxation	Rapid bond rearrangement under activation	[23,89,90,91,92,93,94,95,96,97,98]
Boronic ester exchange	Associative exchange	Often catalyst-free but environmentally regulated	Strong neighboring-group and buffering-ion effects	Highly sensitive to humidity and pH	Enables room temperature exchange	Environment- responsive topology rearrangement	[24,41,99,100,101,102,103,104,105,106,107,108]
Siloxane exchange	Associative siloxane equilibration	Often catalyst- or silanolate- mediated	Strong in amine/amide assisted systems	Sensitive to catalyst and hydroxyl environment	Governs creep suppression and extrusion behavior	Associative rearrangement with preserved connectivity	[25,109,110,111,112,113,114,115,116,117]

**Table 2:** Quantitative comparison of activation energy and operating temperature for representative dynamic covalent thermosets.

Dynamic Chemistry	Representative Activation Energy	Typical Reprocessing Temperature	Ref.
Transesterification	53–148 kJ mol^−1^	140–220 °C	[118,119,120]
Diels–Alder	7.04 kJ mol^−1^ for DA; 57.9 kJ mol^−1^ for retro-DA	retro-DA significant above 100–140 °C	[121,122,123]
Imine Exchange	28–68 kJ mol^−1^	RT to <120 °C	[124,125,126]
Disulfide Exchange	99–357 kJ mol^−1^	RT to ~180 °C	[122,127,128,129]
Boronic ester Exchange	15.9–23.6 kJ mol^−1^ for model metathesis	RT to <120 °C	[122,130,131]
Siloxane Exchange	60–130 kJ mol^−1^	180–250 °C	[31,32,132,133,134]

RT denotes room temperature.

## Data Availability

No new data were created or analyzed in this study.
